# Transition of the Bacterial Community and Culturable Chitinolytic Bacteria in Chitin-treated Upland Soil: From *Streptomyces* to Methionine-auxotrophic *Lysobacter* and Other Genera

**DOI:** 10.1264/jsme2.ME19070

**Published:** 2020-01-11

**Authors:** Yukari Iwasaki, Tatsuya Ichino, Akihiro Saito

**Affiliations:** 1 Department of Materials and Life Science, Shizuoka Institute of Science and Technology, 2200–2 Toyosawa, Fukuroi, Shizuoka 437–8555, Japan

**Keywords:** *Lysobacter*, methionine, chitin, d-Met, *Streptomyces*

## Abstract

Chitin amendment is an agricultural management strategy for controlling soil-borne plant disease. We previously reported an exponential decrease in chitin added to incubated upland soil. We herein investigated the transition of the bacterial community structure in chitin-degrading soil samples over time and the characteristics of chitinolytic bacteria in order to elucidate changes in the chitinolytic bacterial community structure during chitin degradation. The addition of chitin to soil immediately increased the population of bacteria in the genus *Streptomyces*, which is the main decomposer of chitin in soil environments. *Lysobacter*, *Pseudoxanthomonas*, *Cellulosimicrobium*, *Streptosporangium*, and *Nonomuraea* populations increased over time with decreases in that of *Streptomyces*. We isolated 104 strains of chitinolytic bacteria, among which six strains were classified as *Lysobacter*, from chitin-treated soils. These results suggested the involvement of *Lysobacter* as well as *Streptomyces* as chitin decomposers in the degradation of chitin added to soil. *Lysobacter* isolates required yeast extract or casamino acid for significant growth on minimal agar medium supplemented with glucose. Further nutritional analyses demonstrated that the six chitinolytic *Lysobacter* isolates required methionine (Met) to grow, but not cysteine or homocysteine, indicating Met auxotrophy. Met auxotrophy was also observed in two of the five type strains of *Lysobacter* spp. tested, and these Met auxotrophs used d-Met as well as l-Met. The addition of Met to incubated upland soil increased the population of *Lysobacter*. Met may be a factor increasing the population of *Lysobacter* in chitin-treated upland soil.

Chitin, which is a polymer of *N*-acetylglucosamine, is the most abundant renewable natural resource after cellulose (Muzzarelli, 1977; Deshpande, 1986; Gooday, 1991). It is widely distributed in nature, particularly in marine invertebrates, insects, fungi, and algae (Nicol, 1991). The supplementation of soil with chitin effectively suppresses plant-pathogenic fungi (Mitchell and Alexander, 1962; Buxton *et al.*, 1965; Khalifa, 1965; Henis *et al.*, 1967; Sneh and Henis, 1970; Van Eck, 1978; Murakami *et al.*, 2009; Cretoiu *et al.*, 2013) and nematodes (Mian *et al.*, 1982; Sarathchandra *et al.*, 1996). In most, but not all cases, the mechanisms underlying this suppressive activity often involve a change in the structure and/or activity of the microbiota in the soil, which subsequently suppresses plant pathogens (Weller *et al.*, 2002; Mendes *et al.*, 2011). Chitinolytic microorganisms that hydrolyze the chitinous hyphae of pathogenic fungi increase their numbers and activity in response to the chitin added. Alternatively, secondary responders to added chitin may suppress pathogens (Cretoiu *et al.*, 2013).

Previous studies that isolated microbial community members from soils showed that *Streptomyces*, *Micromonospora*, *Nocardia* of *Actinomycetes*, *Achromobacter*, *Flavobacterium*, *Chromobacterium*, *Bacillus*, *Cytophaga*, and *Pseudomonas* were representative genera of chitinolytic bacteria, while *Aspergillus* and *Mortierella* were isolated as chitinolytic fungi (Veldkamp, 1955). 16S rRNA gene analyses revealed that *Actinobacteria* and *Oxalobacteraceae* populations and their ability to suppress plant pathogens increased in chitin-treated soils (Cretoiu *et al.*, 2013). Another study demonstrated that chitin enrichment led to an increase in *Actinobacteria*, γ-*Proteobacteria*, and β-*Proteobacteria*, suggesting the specific selection of these chitin-degrading bacteria (Jacquiod *et al.*, 2013). Various *chiA* genotypes including β-*Proteobacteria*, γ-*Proteobacteria*, *Actinobacteria*, *Acidobacteria*, *Bacteroidetes*, *Firmicutes*, *Planctomycetes*, and *Chloroflexi* were shown to respond to chitin supplementation in soil slurries (Wieczoreck *et al.*, 2014). However, limited information is currently available on temporal changes in the bacterial community structure of chitin-supplemented soil.

We previously investigated the degradation profile of chitin in incubated upland soil and found that added chitin exponentially decreased over time (Kumeta *et al.*, 2018). We herein aimed to identify the chitinolytic bacterial genera involved in exponential chitin degradation using the same soil samples. We performed an amplicon sequencing analysis to investigate the mechanisms by which the bacterial community structure is altered during exponential chitin degradation. We also characterized the chitinolytic bacterial strains isolated from soil samples.

## Materials and Methods

### Preparation of soil samples

We used soil samples prepared for our previous study that was conducted to investigate chitin degradation profiles (Kumeta *et al.*, 2018). The surface soil of Brown Forest soil, classified as light clay, in an upland field was air-dried and passed through a sieve with 2-mm meshes. Chitin powder (Wako Chemicals) was added to an approximate final amount of 60 g kg^–1^ and mixed. The water content was maintained at 60% of the maximum water-holding capacity. A box containing the soil was covered with a lid using pine halls and incubated at 25°C. In the analysis, soil samples were taken from 10 distinct points in the box, mixed, and stored at –‍80°C.

### 16S rRNA gene analysis of the prokaryotic community structure

DNA was extracted from 0.4 g of each soil sample according to a previously reported method (Takada-Hoshino and Matsumoto, 2004) and dissolved in 80 μL of DNase-free water using a FastDNA SPIN Kit for Soil (MP Biomedicals). PCR was performed on extracted DNAs to amplify the V4 region of the 16S rRNA gene using Ex *Taq* DNA polymerase (Takara Bio) and the following primers: 515F (5′-ACACTCTTTCCCTACACGACGCTCTTCCGATCT-GTGCCAGCMGCCGCGGTAA-3′) and 806R (5′-GTGACTGGAGTTCAGACGTGTGCTCTTCCGATCT-GGACTACHVGGGTWTCTAAT-3′) (Caporaso *et al.*, 2011). The PCR products of appropriate sizes were purified using a Wizard SV Gel and PCR Clean-Up System (Promega). PCR fragments (20 ng) were subjected to paired-end sequencing using an Illumina MiSeq platform (Illumina) at Fasmac. Sequences were quality filtered and analyzed with the Quantitative Insights Into Microbial Ecology (QIIME) pipeline (version 1.9.0 [Caporaso *et al.*, 2010]). The remaining sequences (see Table S1 for the detailed numbers of sequences) were clustered into OTUs at the 97% level and classified using the Greengenes 13-8 reference database (Caporaso *et al.*, 2011; McDonald *et al.*, 2012) as implemented in QIIME. Close relatives of representative OTUs were identified by a BLASTn analysis and a phylogenetic analysis using the SILVA database (SILVA SSU Ref NR_128 database) and backbone tree (tree_SSURefNR99_1200slv_128) in the ARB program for the sequence analysis (Ludwig *et al.*, 2004).

### Isolation and classification of microorganisms

Upland soil was incubated for 5 and 45‍ ‍d with powdered chitin (Kumeta *et al.*, 2018). Microbial strains were then isolated from chitin-treated soil by spreading a soil suspension on YG agar plates (0.1% [w/v] glucose, 0.1% [w/v] yeast extract [BD], 0.03% [w/v] dipotassium hydrogen phosphate [K_2_HPO_4_], 0.02% [w/v] KH_2_PO_4_, 0.01% [w/v] MgSO_4_, and 1.5% [w/v] agar [pH 6.8]) followed by an incubation at 30°C for 7 d. Microbial strains were purified by plate streaking. Each of the purified strains was cultivated in YG liquid medium, harvested by centrifugation, and subjected to total DNA preparation using a Quick-DNA Bacterial/Fungal kit (Zymoresearch). 16S rRNA genes were amplified by PCR using KOD FX Neo DNA polymerase (TOYOBO) according to the manufacturer’s instructions with the following primers: 27f (5′-AGAGTTTGATCMTGGCTCAG-3′) and 1522r (5′-AAGGAGGTGATCCAGCCGCA-3′). PCR products were electrophoresed, and DNA fragments of approximately 1,500 bp in length were purified using a Wizard SV Gel and PCR Clean-Up System (Promega). The nucleotide sequences of the purified DNA fragments were first partially elucidated using the fragments as templates with the primers 27f (see above for the nucleotide sequence) and 518r (5′-GTATTACCGCGGCTGCTG-3′) in order to identify the strains obtained briefly. Raw data were analyzed using Genetyx software (Nihongenetics), and nucleotide sequences were elucidated. A homology search was performed with Blast (Altschul *et al.*, 1990) at the DNA Data Bank of Japan (DDBJ). The whole sequences of the amplified 16S rRNA gene fragments originating from the *Lysobacter* strains were elucidated using the purified PCR product as the template. 16S rRNA gene sequences were aligned with default parameters using MEGA v.7.0 software (Kumar *et al.*, 2016). The phylogenetic tree was then constructed with the neighbor-joining method by MEGA v.7.0 software. To evaluate tree topologies, bootstrap values were calculated with 1,000 replicates.

### Chitinolytic evaluation of bacterial isolates

Isolated strains were inoculated into minimal medium (MM) (10‍ ‍mM K_2_HPO_4_, 10‍ ‍mM KH_2_PO_4_, 1‍ ‍mM CaCl_2_, 0.5‍ ‍mM MgCl_2_ supplemented with 0.1% [v/v] trace element solution, and 1.5% [w/v] agar) (Schlochtermeier *et al.*, 1992) containing 0.1% (w/v) colloidal chitin as a carbon source (MMCC medium), as reported previously (Saito *et al.*, 2013) and on MMCC medium supplemented with 0.1% (w/v) yeast extract (YECC medium). Cultures were incubated at 30°C for 10 d. Chitinolytic ability was assessed by the formation of clearing zones around the colonies.

### Evaluating auxotrophy in isolated *Lysobacter* strains

A 1-μL cell suspension of each *Lysobacter* isolate was spotted so that it formed a line on MM agar medium containing 10‍ ‍mM glucose as a carbon source. On one side of the spotted line, 7 μL of 2% (w/v) yeast extract, casamino acid, or one of several amino acids (l-alanine, l-arginine, l-asparagine, l-cysteine, d-glutamic acid, l-glutamine, glycine, dl-homocysteine, l-leucine, l-lysine, d-methionine, l-methionine, l-proline, d-serine, l-threonine, or l-valine) was streaked in a linear manner perpendicular to the spotted line (Fig. S1). Cultures were incubated at 30°C for 7 d, and growth was monitored for the indication of auxotrophy. Five type strains of *Lysobacter* spp. supplied by the Japan Collection of Microorganisms (JCM) (RIKEN, Japan) and the Biological Resource Center, NITE (NBRC, Japan) were used for comparison: *L. rhizosperae* JCM 30321^T^, *L. panacisoli* JCM 19212^T^, *L. niastensis* NBRC 106399^T^, *L. koreensis* NBRC 101156^T^, and *L. concretionis* NBRC 102010^T^.

### PCR primer design for *Lysobacter* spp.

To detect *Lysobacter* in incubated soils, we used the previously reported primers: L4 (5′-GAGCCGACGTCGGATTAGCTAGTT-3′) (Hu, 2010), which was designed to detect *Lysobacter* (Hu, 2010) in soil, and Fw_Lyso_guanxl (non-selective forward primer) (5′-CAACGCGAAGAACCTTACC-3′) and Rev_Lyso_guanxl (selective reverse primer) (5′-TGCAGCACCTGTCTCAC-3′) to analyze the indigenous population of three closely related *Lysobacter* spp. using real-time PCR with a TaqMan probe (Postma *et al.*, 2011).

To design PCR primers for detecting and quantifying *Lysobacter* spp., the 16S rRNA gene sequences of the seven isolated *Lysobacter* strains, as well as 40 strains of *Xanthomonadaceae*, were aligned to identify DNA regions with nucleotide sequences specific for *Lysobacter* spp. (Fig. S2). The specificities of the designed primers were confirmed by PCR using *Lysobacter* spp. total DNA, as described above, with the following strains: *Pseudoxanthomonas* sp. 45-47-2 and 45-43 (isolated from chitin-treated incubated soil in the present study), *P. koreensis* NBRC 101160^T^, *P. taiwanensis* NBRC 101072^T^, *P. japonensis* NBRC 101033^T^, *Xanthomonas campestris* NBRC 13551, *Stenotrophomonas* *nitritireducens* JCM 13311^T^, *Dokdonella immobilis* JCM 15763^T^, and *D. soli* JCM 15421^T^. PCR was conducted using *TaKaRa Ex Taq* DNA polymerase Hot Start Version (Takara Bio). Denaturing, annealing, and extension temperatures were set to 94°C for 15 s, 70.5°C for 30 s, and 72°C for 2 min, respectively.

### Detection and quantification of *Lysobacter* spp. in soil

To investigate whether methionine increases the population of *Lysobacter*, air-dried and sieved Brown Forest soil (see above) was placed in a sterile polypropylene tube, and its water content was adjusted to 60% of its maximum water-holding capacity with sterilized MilliQ water supplemented with or without l-methionine (final 0.1% [w/w]). The same concentration of glucose was added to another soil sample for comparison. After an incubation at 25°C, total DNA was extracted from 0.4 g of each soil sample according to a previously reported method (Takada-Hoshino and Matsumoto, 2004) and dissolved in 80 μL of DNase-free water using a FastDNA SPIN Kit for Soil (MP Biomedicals). Regarding the detection and quantification of *Lysobacter* spp., PCR was performed using the 16S_Lyso_F1 and 16S_Lyso_R1 primers (see Results and Discussion for details). After end-point PCR, which was performed under the conditions described (see above), the products were electrophoresed on agarose gels and visualized by staining with ethidium bromide. Regarding the quantification of DNA, an Applied Biosystems 7500 Fast Real-Time PCR System (Thermo Fisher Scientific) was used with SYBR Green and TB GreenTM *Premix Ex Taq* TM II (Takara Bio). Using the total DNA of *L. panacisoli* JCM 19212T as the template, the corresponding PCR product obtained was used as the standard.

### Nucleotide sequence accession numbers

The 16S rRNA gene sequences of the seven *Lysobacter* isolates, as well as two *Pseudoxanthomonas* strains isolated in the present study, have been deposited in the DDBJ under accession numbers LC481367-LC481375. Illumina sequencing data were deposited in the DDBJ/ENA/GenBank database under BioProject ID PRJDB8756 and BioSample ID SAMD00185699–SAMD00185712.

## Results and Discussion

### Bacterial community structure fluctuations in incubated upland soil supplemented with chitin

**Fig. 1.**
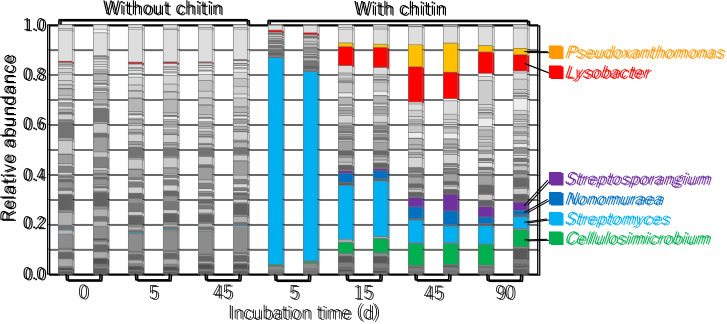
Bacterial community structure during the incubation of upland soil treated with or without chitin powder. A section of the 16S rRNA gene from the DNA originating from each soil sample was amplified and subjected to an amplicon sequencing analysis. The community composition of each sample is shown at the genus level. *Streptomyces*, *Lysobacter*, *Streptosporangium*, *Nonomuraea*, *Cellulosimicrobium*, and *Pseudoxanthomonas* amounts are highlighted in light blue, red, purple, blue, green, and orange, respectively.

After incubating chitin-treated soil samples at 25°C for 5 d, approximately 80% of the bacterial community consisted of the genus *Streptomyces* (Fig. 1), the replication of which markedly increased in chitin-supplemented soil (Mitchell and Alexander, 1962; Sneh and Henis, 1970). As the population of *Streptomyces* decreased, those of *Pseudoxanthomonas* and *Lysobacter* bacteria increased (Fig. 1). Bacteria belonging to the genera *Cellulosimicrobium*, *Streptosporangium*, and *Nonomuraea* also increased following the decrease in the population of *Streptomyces*. Among the genera with populations that increased in incubated chitin-supplemented upland soil, strains of *Streptomyces*, *Lysobacter*, *Cellulosimicrobium*, and *Streptosporangium* exhibited chitin-degrading activity and extracellular chitinase production (Deshpande, 1986; Fleuri *et al.*, 2009; Qian *et al.*, 2012; Shirlin and Kalaiarasi, 2016). They also possessed several putative chitinase genes (CAZy: http://www.cazy.org [Henrissat and Davies, 1997]) (Table S2), suggesting the ability to utilize chitin as carbon and nitrogen sources. In contrast, the chitin degradation activities of *Nonomuraea* and *Pseudoxanthomonas* strains currently remain unknown. The populations of these genera may utilize chitin degradation products generated by the extracellular chitinases produced by chitinolytic microorganisms. However, genome sequence analyses revealed the presence of putative chitinase genes on the chromosomes of *N. gerenzanensis* ATCC 39727, *N.* sp. ATCC 55076, and *P. suwonensis* J1 (seven, six, and one putative genes, respectively) (CAZy) (Table S2), demonstrating the potential chitinolytic activity of some strains of *Nonomuraea* and *Pseudoxanthomonas*.

### Populations of chitinolytic bacteria in chitin-added upland soil

To evaluate the community of culturable chitin-degrading bacteria in chitin-added soil, 83 and 79 (total of 162) strains were isolated, using non-selective agar medium, from chitin-supplemented soils after an incubation at 25°C for 5 and 45 d, respectively. On MMCC agar medium, which contains colloidal chitin as a carbon source, 59 (71%) and 27 (37%) strains isolated from the 5- and 45-day-incubated soil samples, respectively, exhibited chitin degradation. On YECC agar medium, which contains yeast extract in addition to colloidal chitin, 69 (83%) and 35 (45%) strains isolated from the 5- and 45-day-incubated soil samples, respectively, showed chitin degradation. Therefore, the number of chitin-degrading isolates was higher in 5-day-incubated chitin-added soil than in 45-day-incubated soil. This is consistent with previous findings showing that chitinase activity was higher in 5-day-incubated soil than in 45-day-incubated soil (Kumeta *et al.*, 2018). The greater number of chitin-degrading strains observed on YECC than on MMCC suggests that some of the strains are auxotrophs.

**Fig. 2.**
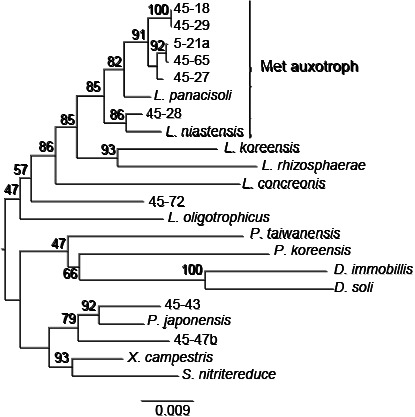
Phylogenetic positions of *Lysobacter* strains isolated in this study. The 16S rRNA genes of the seven *Lysobacter* and two *Pseudoxanthomonas* strains isolated in this study were aligned with those of neighboring species and genera listed in Table 3, and phylogenetic relationships were calculated by the neighbor-joining method. The bar with the number 0.009 indicates the number of substitutions per site. The number at each node shows the result of 1,000 bootstrap analyses as a percent (%). 5-21a, 45-18, 45-27, 45-28, 45-29, 45-65, and 45-72 were strains of *Lysobacter* isolated in this study; 45-43 and 45-47b were strains of *Pseudoxanthomonas* isolated in this study. *L.* signifies *Lysobacter*; *P.*, *Pseudoxanthomonas*; *D.*, *Dokdonella*; *X.*, *Xanthomonas*; and *S.*, *Stenotrophomonas*. See Table 3 for the names of the reference strains.

Partial 16S rRNA gene sequences were elucidated for 161 out of the 169 strains isolated from chitin-added incubated upland soil using a non-selective medium. Seven of the identified strains belonged to the genus *Lysobacter*. Six out of the seven *Lysobacter* strains exhibited chitin-degrading activity on YECC agar medium, suggesting the involvement of chitinolytic *Lysobacter* strains in chitin degradation in incubated upland soil. One strain (5-21a) was isolated from 5-day-incubated soil, while the other 6 strains were obtained from 45-day-incubated soil. In contrast, 46 and 9 chitinolytic strains isolated from 5- and 45-day-incubated soil were identified as *Streptomyces* (unpublished data). These results were consistent with those of the bacterial community structure analysis showing that the numbers of *Lysobacter* and *Streptomyces* bacteria were higher and lower, respectively, in 45-day-incubated soil than in 5-day-incubated soil (Fig. 1). Amplified 16S rRNA gene sequences (approximately 1.4‍ ‍kb) from the seven *Lysobacter* isolates revealed that 5 strains (5-21a, 45-18, 45-27, 45-29, and 45-65) were closely related to *L. soli* (99.3–100%) (Srinivasan *et al.*, 2010), strain 45-28 was closely related to *L. niastensis* NBRC106399^T^ (99.0%) (Weon *et al.*, 2007), and strain 45-72 was closely related to *L. dokdonensis* DS-58 (99.9%) (Oh *et al.*, 2011). Strain 45-72, which did not exhibit chitinolytic activity, was phylogenetically apart from the other six *Lysobacter* isolates (Fig. 2). The six chitinolytic *Lysobacter* strains did not significantly grow on MMCC agar medium, but still grew well, indicating chitinolytic activity. Since we suspected auxotrophy, we investigated the effects of nutritional characteristics on the growth of the strains.

### Methionine auxotrophy of isolated *Lysobacter* strains

*Lysobacter* sp. strain 5-21a isolated from incubated chitin-supplemented soil did not significantly grow on minimal media supplemented with colloidal chitin or glucose as a carbon source. However, it grew in the presence of yeast extract or casamino acid (Fig. S1 and Table 2). These results suggest that *Lysobacter* sp. 5-21a is an amino acid auxotroph. Among the 16 amino acids tested, only l- or d-methionine (Met) rescued the growth of 5-21a on minimal agar medium supplemented with glucose (Table 2), demonstrating Met auxotrophy. Among the six other isolates of *Lysobacter*, five strains (45-18, 45-27, 45-28, 45-29, and 45-65) also demonstrated Met auxotrophy, while one (45-72) did not (Table 2). 45-72, which is phylogenetically apart from the other six *Lysobacter* strains isolated in the present study (Fig. 2), may be an auxotroph against another amino acid. Among the five type strains of *Lysobacter* tested, *L. panacisoli* and *L. niastensis*, which are closely related to the five Met auxotroph *Lysobacter* isolates (Fig. 2), also indicated Met auxotrophy, while the three other species (*L. rhizospherae*, *L. koreensis*, and *L. concretionis*) did not (Table 2). The growth of *L. rhizosphaerae*, similar to *Lysobacter* sp. 45-72, was enhanced by casamino acid, but not by Met, and casamino acid did not enhance the growth of *L. koreensis* or *L. concretionis*. These results indicate that Met auxotrophy observed in the *Lysobacter* isolates was shared with some type strains of *Lysobacter* spp.

The growth of *Lysobacter* sp. 5-21a was not rescued by cysteine or homocysteine (Table 2). Met auxotroph *Lysobacter* strains were not able to utilize l-Met or d-Met as a sole carbon source (data not shown). 5-21a does not appear to have the capacity to synthesize or metabolize Met via these amino acids and may use Met only for anabolism, such as protein synthesis. It is important to note that Met auxotroph *Lysobacter* strains, including two type strains, utilize d-Met as well as l-Met (Table 2); however, the purity of commercially obtained d-Met needs to be examined. *Pseudomonas putida* IFO 12996 produces broad specificity amino acid racemase (BAR) (E.C. 5.1.1.10) (Soda and Osumi, 1969). *Vibrio cholerae* also produces a periplasmic broad spectrum racemase (BsrV) that racemizes several types of amino acids (Lam *et al.*, 2009; Espaillat *et al.*, 2014). BAR and BsrV racemize d-Met and other amino acids (Kino *et al.*, 2007; Espaillat *et al.*, 2014). *Lysobacter* strains may produce these types of enzymes to utilize d-Met as a Met source.

### PCR primer design for *Lysobacter* spp.

**Fig. 3.**
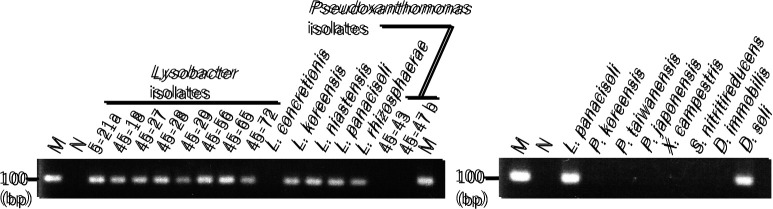
Amplification of a section of the 16S rRNA gene using primers 16S_Lyso_F1 and 16S_Lyso_R1 designed in this study. The total DNA of each bacterial strain was used as the template during PCR. See Materials and Methods for detailed PCR conditions. M signifies the DNA size marker; N, negative control (without a DNA template); *L.*, *Lysobacter*; *P.*, *Pseudoxanthomonas*; *X.*, *Xanthomonas*; and *D.*, *Dokdonella*. The size of the DNA marker is indicated in base pairs (bp)

In order to select PCR primers for investigating the effects of Met treatments on the population of *Lysobacter* in soil, we initially demonstrated the specificities of the primers reported previously, and found that the primer set L4 (Hu, 2010) and another universal bacterial primer 518R, as well as Fw_Lyso_guanxl and Rev_Lyso_guanxl (Postma *et al.*, 2011), amplified the corresponding DNA fragment from the total DNAs of *Lysobacter* spp. and strains from the genus *Pseudoxanthomonas* (Table 3. See Fig. 2 for their phylogenetic positions). Therefore, we designed a new primer set (16S_Lyso_F1 [5′-CGGGTTGTAAAGCWCTTTTGTCC-3′] and 16S_Lyso_R1 [5′-GAAGTTAGCCGGTGCTTATTCTTCC-3′]) based on the alignment of 16S rRNA genes (Fig. S2) that was more specific against *Lysobacter*. The new primer set successfully amplified DNA fragments of the expected sizes in all *Lysobacter* strains tested, except for* L. concretionis* (Fig. 3), the nucleotide sequence of which does not match the 3′-end of 16S_Lys_F1 (Fig. S2). In contrast, the primer set did not amplify the 16S rRNA gene from closely related genera including *Pseudoxanthomonas*, *Xanthomonas*, and *Stenotrophomonas* when using total DNA. However, the primer set also amplified the corresponding DNA fragment from the total DNA of *D. soli*, the nucleotide sequence of which matches those of the primers. However, it did not amplify DNA fragments from *D. immobilis* (Fig. 3 and S2). Thus, we concluded that the designed primer set was more specific for detecting *Lysobacter* spp. than those reported to date, and also amplified the corresponding gene of at least *D. soli*.

### Ability of the designed PCR primer set to quantitatively evaluate *Lysobacter* populations

**Fig. 4.**
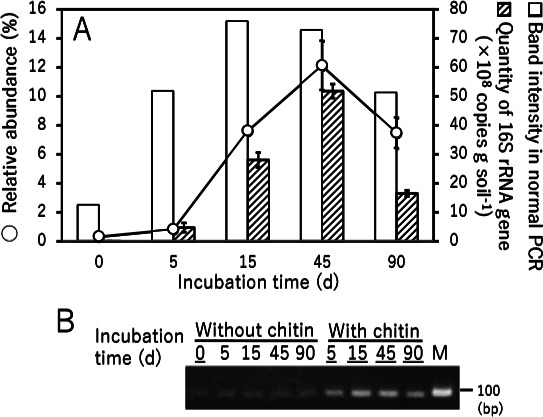
*Lysobacter* population evaluation in incubated chitin-treated upland soil. (A) Open circles signify the relative abundance of the sequences assigned to the genus *Lysobacter* in the bacterial community structure analysis shown in Fig. 1; white boxes, the amount of PCR product amplified with the primers 16S_Lyso_F1 and 16S_Lyso_R1; textured boxes, the number of 16S rRNA gene fragment copies detected by the primers 16S_Lyso_F1 and 16S_Lyso_R1 using real-time PCR. (B) The products of normal PCR were electrophoresed on an agarose gel and stained with ethidium bromide. The intensities of the bands corresponding to the PCR products were measured. M signifies the DNA size marker (100 bp). Samples with band intensities indicated in panel A are underlined.

The ability of the PCR primer set to evaluate the population of *Lysobacter* was confirmed by both semi-quantitative (normal) PCR and quantitative PCR (qPCR) using DNA samples prepared for the amplicon sequencing analysis (Fig. 1) as templates. Normal PCR results were consistent with the amplicon sequencing analysis of *Lysobacter* populations (*i.e.* the amount of the PCR product was lower in soil without added chitin, but increased in chitin-supplemented soil) (Fig. 4). Therefore, a positive correlation appears to exist between the amount of the PCR product and the amount of *Lysobacter* in chitin-supplemented soil. The results of the qPCR analysis using SYBR green were also consistent with the amplicon sequencing analysis, indicating the utility of the primer set at quantifying *Lysobacter* populations (Fig. 4). Thus, we concluded that the primers 16S_Lyso_F1 and 16S_Lyso_R1 are appropriate for evaluating the quantity of *Lysobacter* species, even though they also amplify the corresponding gene in some species of the genus *Dokdonella*.

### *Lysobacter* population increases in Met-supplemented incubated upland soils

**Fig. 5.**
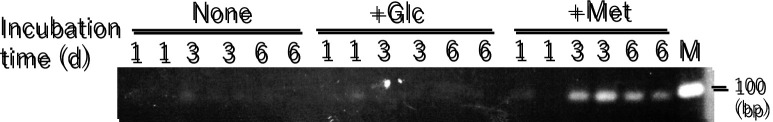
Effects of methionine on *Lysobacter* populations in incubated upland soil. The *Lysobacter* population was evaluated by normal PCR using the primers 16S_Lyso_F1 and 16S_Lyso_R1. PCR products were electrophoresed on an agarose gel and stained with ethidium bromide. M signifies the DNA size marker (100 bp); None, without a supplement; +Glc, supplemented with glucose; +Met, supplemented with methionine. Incubated soil was prepared in duplicate for each treatment.

The amount of the PCR product amplified with the 16S_Lyso_F1 and 16S_Lyso_R1 primers increased when DNA templates prepared from incubated upland soil supplemented with 0.1% d,l-Met were examined; however, it did not increase in non-supplemented soil or soil supplemented with 0.2% glucose (Fig. 5). These results strongly suggest that the population of *Lysobacter* increased due to the addition of d,l-Met. An increase in the *Lysobacter* spp. population by Met was already observed on a partial-nitritation biofilter for wastewater treatment (Gonzalez-Martinez *et al.*, 2016). The findings obtained suggested that *Lysobacter* spp. does not grow due to methionine consumption itself, but due to predation of the bacterial biomass in the system (Gonzalez-Martinez *et al.*, 2016) because *Lysobacter* spp. has been identified as heterotrophic with lytic activity against other microorganisms, including fungi and nematodes (Christensen and Cook, 1978). The present results suggesting the Met auxotrophy of *Lysobacter* strains imply that the increased *Lysobacter* spp. population on the partial nitritation biofilter may be a Met auxotroph.

*Lysobacter* spp. were isolated from clay soils and not from sandy soils found in the arable fields of organic farms in the Netherlands (Postma *et al.*, 2008). A real-time PCR analysis demonstrated that populations of *L. antibioticus*, *L. capsica*, and *L. gummosus* were approximately 6.22–6.95 log gene copies g of soil^–1^ larger in clay soils from arable fields of organic farms than in sandy soils (Postma *et al.*, 2011). We herein used light clay soil from an upland field in Japan (see Materials and Methods for details). Met may be useful for increasing and/or activating indigenous *Lysobacter* spp. in soils; however, the presence of *Lysobacter* spp. and its growth modulation by chitin or Met need to be investigated in soils of different textures.

In the present study, we demonstrated an immediate increase and subsequent decrease in *Streptomyces* (Fig. 1), one of the main decomposers of chitin in soil. As the population of *Streptomyces* decreased over time, *Lysobacter*, *Pseudoxanthomonas*, *Cellulosimicrobium*, *Streptosporangium*, and *Nonomuraea* populations increased (Fig. 1). We isolated six *Lysobacter* strains exhibiting chitin-degradation activity from chitin-supplemented soil, suggesting that *Lysobacter* was involved in chitin degradation in chitin-treated upland soil. *Lysobacter* isolates were Met auxotrophs. The population of *Lysobacter* increased in Met-treated incubated soils (Fig. 5). We assume that Met is a factor contributing to increases in the population of *Lysobacter* in chitin-added upland soil. In a study using synthetic syntrophic microbial communities, biosynthetically costly amino acids, including Met, lysine, isoleucine, arginine, and aromatics, were found to more likely promote stronger cooperative interactions than amino acids that are cheaper to produce (Mee *et al.*, 2014). Met may be a key compound that triggers the transition from *Streptomyces* to *Lysobacter*, and possibly other bacterial genera in chitin-treated upland soil, as the dominant bacterial population. This hypothesis is currently being investigated.

## Supplementary Material

Supplementary Material

## Figures and Tables

**Table 1.** Chitinolytic activities of bacterial strains isolated from chitin-treated upland soil.

Incubation time of chitin-treated upland soil (d)	Number of microbial isolates*	Number of strains exhibiting chitinolytic activity (% against the number of isolates)	
		on MMCC**	On YECC**
5	83 (100)	59 (71)	69 (83)
45	79 (100)	27 (37)	35 (45)

* Microbial strains were isolated using YG agar medium containing yeast extract and glucose (see Materials and Methods for a detailed composition). ** MMCC, minimal agar medium containing 0.1% (w/v) colloidal chitin as a carbon source; YECC, MMCC supplemented with 0.1% (w/v) yeast extract.

**Table 2.** Effects of amino acids on the growth of *Lysobacter* strains on minimal agar medium supplemented with glucose.

	Isolates of *Lysobacter* spp.				Type strains of *Lysobacter* spp.				
	5-21a	45-18, 45-27, 45-28, 45-29, 4-65	45-72		*L. panacisoli* JCM 19212^T^	*L. niastensis* NBRC 106399^T^	*L. rhizospherae* JCM 30321^T^	*L. koreensis* NBRC 101156^T^	*L. concretionis* NBRC 102010^T^
Casamino acid	+	+	+		+	+	+	–	–
l-Met	+	+	–		+	+	–	–	–
d-Met	+	+	–		+	+	–	–	–
Other amino acids*	–	nt	nt		nt	nt	nt	nt	nt

* l-alanine, l-arginine, l-asparagine, l-cysteine, d-glutamic acid, l-glutamine, glycine, d,l-homocysteine, l-leucine, l-lysine, l-proline, d-serine, l-threonine, or l-valine. +, growth was significantly enhanced; –, growth was not significantly enhanced; nt, not tested.

**Table 3.** Amplification of target DNA fragments

Species	Strain	Primers (References)		
		L4 (Hu, 2010) 518r	Fw_Lyso_guanxl Rev_Lyso_guanxl (Postma *et al.*, 2011)	16S_Lyso_F1 16S_Lyso_R1 (This study)
*L. koreensis*	NBRC101156^T^	+	–	+
*L. rhizosphaerae*	JCM30321^T^	+	–	+
*L. concretionis*	NBRC102010^T^	+	+	–
*L. niastensis*	NBRC106399^T^	+	+	+
*L. panacisoli*	JCM19212^T^	+	+	+
*L. oligotrophicus*	JCM18257^T^	+	+	+
*Lysobacter* sp.	5-21a*	+	+	+
	45-65*	+	+	+
	45-27*	+	+	+
	45-18*	+	+	+
	45-29*	+	+	+
	45-28*	+	+	+
	45-72*	+	+	+
*Pseudoxanthomonas* sp.	45-43*	+	–	–
*Pseudoxanthomonas* sp.	45-47b*	+	+	–
*Pseudoxanthomonas japonensis*	NBRC101033^T^	nt	nt	–
*Pseudoxanthomonas koreensis*	NBRC101160^T^	nt	nt	–
*Pseudoxanthomonas taiwanensis*	NBRC101072^T^	nt	nt	–
*Stenotrophomonas nitritireducens*	JCM13311^T^	nt	nt	–
*Xanthomonas campestris*	NBRC13551	nt	nt	–
*Dokdonella immobilis*	JCM15763^T^	nt	nt	–
*Dokdonella soli*	JCM15421^T^	nt	nt	+

* Strains isolated in this study. +, amplified; –, not amplified; nt, not tested.
