# Supplementary material for: Transition of the Bacterial Community and Culturable Chitinolytic Bacteria in Chitin-treated Upland Soil: From Streptomyces to Methionine-auxotrophic Lysobacter and Other Genera
Source: Microbes Environ. 2020 Jan 11;35(1):ME19070. doi: 10.1264/jsme2.ME19070 (PMC7104288; doi:10.1264/jsme2.ME19070)
Supplement: Supplementary file 1 — Supplementary Material [file 35_19070_s1.pdf]

Supplementary Materials

Table S1. Numbers of quality filtered sequences and OTUs obtained by the amplicon sequencing analysis targeting 16S rRNA gene

Treatment of soil	Incubation time (d)	Numbers of quality filtered sequences obtained	Numbers of OTUs
None	0	14,296	500
		15,820	508
	5	21,425	472
		39,536	532
	45	26,607	508
		16,737	451
+Chitin	5	19,455	272
		26,142	328
	15	17,734	342
		19,079	350
	45	16,710	308
		11,631	284
	90	30,947	338
		12,561	306

Table S2. Number of putative chitinase genes in strains of *Lysobacter, Cellulosimicrobium, Nonomuraea, Pseudoxanthomonas, Streptomporangium,* and *Streptomyces*, which increased in the chitin-treated upland soil

Genus	Species	Strain	Numbers of putative chitinase genes	
			GH18*	GH19*
Lysobacter	*enzymogenes*	C3, M497-1, YC36	3	3
	capsici	55, KNU-14	3	2
	antibioticus	76	3	4
		ATCC 29479	3	3
	gumosus	3.2.11	3	5
	maris	HZ9B	1	1
Cellulosimicrobium	*cellulans*	PSBB019	9	3
	sp.	T20	5	3
Nonomuraea	*gerenzanensis*	ATCC 39727	7	0
	sp.	ATCC 55076	6	0
Pseudoxanthomonas	*suwonensis*	J1	1	0
Streptosporangium	*roseum*	DSM 43021	11	2
	sp.	caverna	9	1
Streptomyces	*coelicolor* A3(2)	M145	11	2
	griseus	NBR 13350	8	2
	lividans	TK24	11	2
	avermitilis	MA-4680 (NBRC 14893)	8	3

The numbers of putative chitinase genes in each bacterial strains were excerpted from CAZy {http://www.cazy.org (Henrissa and Davies, 1997)}. GH18 indicates family 18 of glycoside hydrolase classification; GH19, family 19 of glycoside hydrolase classification (1).

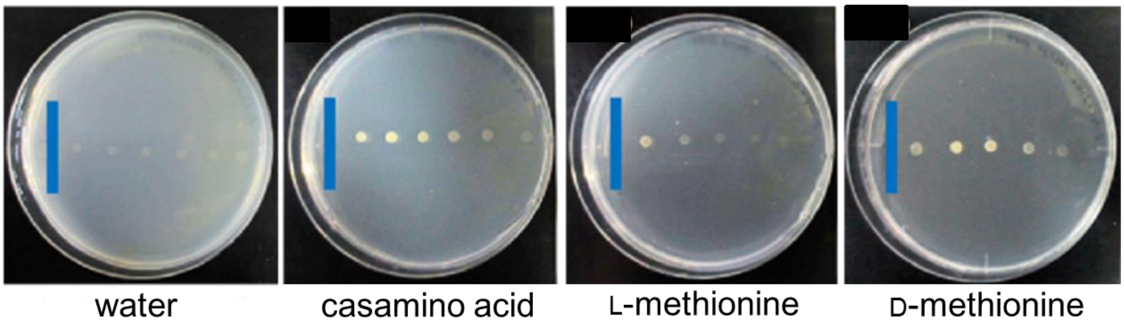

Fig. S1. Effects of casamino acid, L-methionine and D-methionine on the growth of *Lysobacter* sp. strain 5-21a on a minimal agar medium supplemented with glucose as a carbon source. 2.0% casamino acid, L-methionine or D-methionine dissolved in water was streaked linearly and the cells of *Lysobacter* sp. strain 5-21a suspended in sterilized water were spotted vertically against the streaked line of the supplement. The pictures were taken after the incubation at 30ºC for 7 d. Thick blue lines indicate the positions where water, casamino acid, L-methionine, or D-methionine were streaked, respectively.

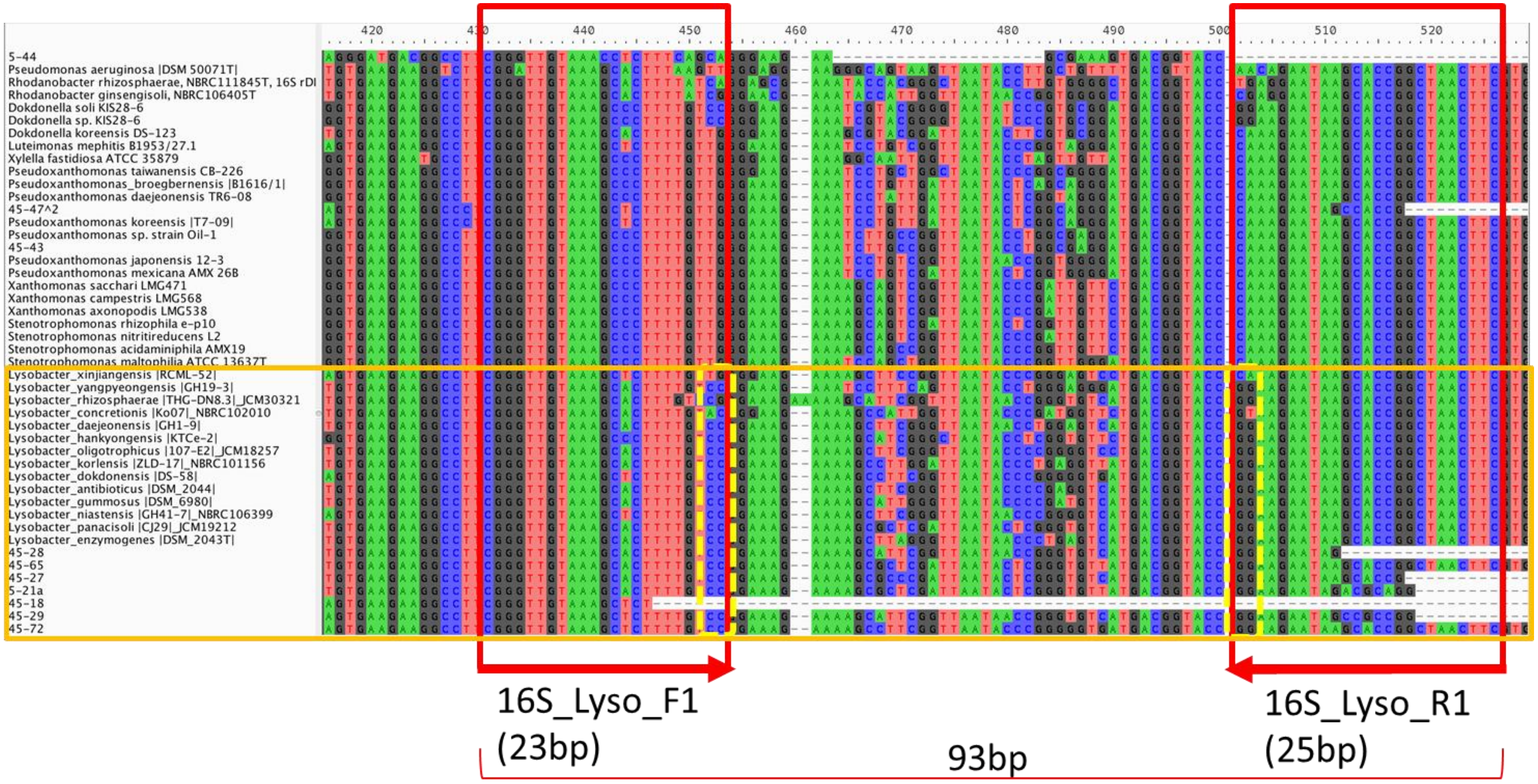

Fig. S2. Alignment of nucleotide sequences of a part of 16S rRNA gene of the strains of the genus *Lysobacter* and the relative genera. The nucleotides A, G, C and T are shown on the green, black, blue and red backgrounds, respectively. The sequences of *Lysobacer* spp. are boxed by yellow line. Two red arrows indicate positions of the designed primer 16S_Lyso_F1 and 16S_Lyso_R1, respectively. The size of the PCR product is also shown. The parts corresponding to the primers are boxed with red lines.

Reference

Henrissat, B., and Davies, G.J. (1997) Structural and sequence-based classification of glycoside hydrolases. *Curr Opin Struct Biol* **7:** 637-644.
